# Analysis of differences in tobacco leaf microbial communities after redrying in Chinese provinces and from abroad

**DOI:** 10.1186/s13568-023-01580-5

**Published:** 2023-08-01

**Authors:** Yifan Zhang, Qiang Xu, Mengmeng Yang, Yue Yang, Jincun Fu, Chenlin Miao, Guiyao Wang, Liwei Hu, Zongyu Hu

**Affiliations:** 1 China Tobacco Jiangsu Industrial Co., Ltd., Nanjing, 210000 Jiangsu China; 2 Zhengzhou Tobacco Research Institute of CNTC, Zhengzhou, 450001 Henan China

**Keywords:** Microbial community, Tobacco aging, 16S rRNA sequencing, Threshed and redried tobacco leaves

## Abstract

Microorganisms play an important role in the tobacco aging process. Before the aging process, raw tobacco leaves must be threshed and redried. In order to explore the differences of microbial community structure of threshed and redried tobacco leaves from different origins at home and abroad, 14 groups of tobacco leaves from 8 different countries were tested by high-throughput DNA sequencing and microbiology analysis. Then, through amplicon sequence variants (ASV) cluster analysis, Venn diagram and species labeling and other microbial diversity analysis, the dominant bacteria and fungi on the surface of threshed and redried tobacco leaves were obtained. The results showed that there were significant differences in the composition of tobacco bacteria and fungi after threshing and redrying from different geographical areas. The relative abundance of *Microbacterium* and *Sphingomonas* in domestic tobacco leaves was significantly higher than that of foreign tobacco leaves. The relative abundance of *Pseudomonas* in foreign tobacco bacterial colonies was significantly higher than that of domestic tobacco leaves. In terms of fungi, the relative abundance of *Aspergillus* and *Alternaria* in domestic tobacco leaves was significantly higher than that of foreign tobacco leaves. *Septoria*, *Sampaiozyma*, *Cladosporium* and *Phoma* account for significantly higher proportions of foreign tobacco leaves. These microorganisms may be indispensable in aging process to form different flavors of tobacco leaves. It provides an important theoretical basis for the further use of microorganisms to promote tobacco leaf aging.

**Supplementary Information:**

The online version contains supplementary material available at 10.1186/s13568-023-01580-5.

## Key points

- Different growth locations of tobacco at home and abroad affect bacterial and fungal communities.
- Proteobacteria and Actinomycetes were the dominant phylum in redrying tobacco leaves.

**Supplementary Information:**

The online version contains supplementary material available at 10.1186/s13568-023-01580-5.

## Introduction

The aging process is the industrial modulation of tobacco leaves before the formulation is used, and is an important link in cigarette production. Before aging process, raw tobacco leaves (TLs) need to go through the process of threshing and redrying. The microorganisms on the surface of tobacco leaves after threshing and redrying play an important role in the aging process of tobacco leaves and were of great significance for improving the quality of tobacco leaves (Di Giacomo et al. 2007). Microbial metabolism can promote the conversion of macromolecules such as protein, starch and cellulose in tobacco leaf substrate, thereby increasing the content of aromatic compounds (Maldonado-Robledo et al. 2003). *Bacillus*, *Pseudomonas*, and *Sphingomonas* were identified as the predominant bacterial populations in flue-cured tobacco (Huang et al. 2010; Zhou et al. 2020). These bacteria can degrade macromolecules in tobacco leaves during aging process and reduce the contents of harmful ingredients such as nicotine (Liu et al. 2015) and tobacco-specific nitrosamines (Vigliotta et al. 2007). For example, *Pseudomonas* sp. ZUTSKD was found to degrade nicotine in tobacco leaves (Wang et al. 2004). The richness of bacterial community on the surface of tobacco leaves was higher than that of fungal community. *Aspergillus*, *Phoma*, *Alternaria* and *Cladosporium* are the main fungal groups in tobacco (Huang et al. 2010; Zhou et al. 2020, 2022; Sajid et al. 2023).

Traditional methods for the separation and purification of tobacco microorganisms on the surface of tobacco leaves have a large workload and long time, and many tobacco microorganisms cannot be cultured under laboratory conditions, so they cannot fully and accurately reflect the structure of foliar microbial communities (Hugenholtz et al. 1998; Zhao et al. 2007). With the development of science and technology, high-throughput sequencing technology has the advantages of large throughput, short cycle and multiple data generation, which can more comprehensively and accurately reflect the microbial community structure on the surface of tobacco leaves, and its application in tobacco microbial research is also increasing. High-throughput sequencing technology, through sequencing the 16SrDNA/18SrDNA/ITS sequences, can simultaneously detect the dominant species, rare species and some unknown species in the sample, obtain the microbial community composition and relative abundance in the sample, which is widely used in the study of plant, soil and intestinal microbial composition (Jo et al. 2020; Nilsson et al. 2019; Wang et al. 2018a). The microorganisms on the surface of tobacco leaves are closely related to the germplasm resources, varieties, origins and grades of tobacco leaves. Studies have found that in tobacco leaves with good quality and aroma quality, there are more types and quantities of microorganisms (Zhang et al. 2019; Ye et al. 2021). However, there are few studies on the differences in microbial community in tobacco leaves from different origins at home and abroad.

In this study, high-throughput sequencing technology was used to study the microbial community composition and diversity of redried tobacco leaf samples from 14 different production areas at home and abroad. This study aims to elucidate the effects of different regions on the surface microorganism of redried tobacco leaves, explore the dominant microbial genus, and provide a theoretical basis for the application of exogenous microbial preparations in the raw tobacco aging process.

## Materials and methods

### Sample collection

A total of 7 domestic geographical areas of tobacco leaves are listed in Table 1, the grade of all the samples was C3F, and the C3F grade is represented by the 7th and 8th top leaf. The list of foreign tobacco leaves is listed in Table 1. A rough location of tobacco leaf samples on the world map was shown in Additional file 1: Fig. S1. Tobaccos were grown and harvested normally in various production areas, and redried in local tobacco redrying factory. The samples were taken at the end of the threshing and redrying process and stored in the refrigerator at − 80 ℃ for high-throughput sequencing.

**Table 1:** Overview of domestic and foreign tobacco leaf samples

Sample name	Place of origin	Tobacco grade	Variety
L4C	Yunnan Lufeng	C3F	Yunyan87
B4C	Guizhou Bozhou	C3F	Yunyan87
N4C	Fujian Ninghua	C3F	CB-1
H4C	Sichuan Huidong	C3F	Yunyan87
G4C	Hunan Guiyang	C3F	Yunyan87
M4C	Henan Mianchi	C3F	Qinyan96
F4C	Shandong Feixian	C3F	Zhongyantexiang301
AR	Argentina	ASBFO	–
US	United States	B1FR	–
ZW	Zimbabwe	L1OA	–
BR	Brazil	B1O	–
MW	Malawi	FLOAT	–
TZ	Tanzania	L1OFT	–
ZM	Zambia	L1M	–

The C3F grade is represented by the 7th and 8th top leaf. Tobacco leaf samples from other countries are mixed tobacco leaves, so the tobacco variety is unclear

*ASBFO* upper, orange, grade 1, *B1FR* upper 2nd shed, orange red, grade 1, *L1OA* upper 2nd shed, orange, grade 1, *B1O* upper 2nd shed, orange, grade 1, *FLOAT* upper, orange, *L1OFT* upper, orange, grade 1, *L1M* upper 2nd shed, orange, grade 1

### DNA extraction, PCR amplification and sequencing

The genomic DNA of the fourteen redried tobacco samples was extracted using the Ezup Column Fungi Genomic DNA Purification Kit (Sangon Biotech, Shanghai, China). The V5–V7 region of the bacteria 16S ribosomal RNA gene was amplified by PCR (94 °C for 5 min, followed by 34 cycles at 94 °C for 1 min, 57 °C for 45 s, and 72 °C for 1 min and a final extension at 72 °C for 10 min) using primers 799F (5′-AACMGGATTAGATACCCKG-3′) and 1193R (5′-ACGTCATCCCCACCTTCC-3′). The ITS1-1F region of the fungal endogenous transcription spacer (ITS1) was amplified by PCR using primers ITS1-1F-F (5′-CTTGGTCATTTAGAGGAAGTAA-3′) and ITS1-1F-R (5′-GCTGCGTTCTTCATCGATGC-3′), PCR reactions were performed in triplicate. Each reaction consisted of a 50 μL mixture containing 25 μL of 2 × Phusion^®^ High-Fidelity PCR Master (Thermo Fisher, Waltham, US), 3 μL of each primer (10 μM), and 10 ng (4 μL) of template DNA. The amplified products were purified, quantified and homogenized to form a sequencing library, and the established library was first inspected for library quality, and the library that passed the quality inspection was sequenced with Illumina HiSeq PE2500 (Illumina, San Diego, US).

### Processing of sequencing data

Raw reads from the original DNA fragments were merged and quality-filtered to obtain Clean Data using FLASH (Magoč and Salzberg 2011) and QIIME (Caporaso et al. 2010). Amplicon sequence variants (ASVs) were clustered with a ≥ 97% similarity cutoff using DADA2 (Li et al. 2020). Next, the representative gene sequences of each ASV were annotated with taxonomic information to obtain the corresponding species information and species-based abundance distribution (Callahan et al. 2017). The taxonomic information annotation of bacterial gene sequences against the 16S ribosomal RNA gene (16S rRNA) database using the RDP classifier (Rognes et al. 2016). Fungi gene sequences were annotated with taxonomic information using the Unite ITS database against each representative sequence (Kõljalg et al. 2013). Compare the standard serial number with the sample with the least number of sequences to normalize the ASV abundance. Chao and Shannon values were calculated at the ASV level using QIIME to reflect the Alpha diversities of bacteria and fungi.

### Availability of data

Data concerning the samples included in this study are deposited in the NCBI BioProject database under BioProject accession number PRJNA970923 and PRJNA971330.”

## Results

### Sequence statistical analysis

A total of 1,848,689 bacterial and 3,218,711 fungal reads were obtained through Illumina sequence analysis. After removing inauthentic data, 1,770,991 valid bacterial (Additional file 2: Table S1) and 2,583,028 fungal reads were obtained from the fourteen samples (Additional file 2: Table S2). The bacterial and fungal valid reads were clustered into 6015 and 3573 ASVs, respectively. The Chao indices of bacteria varied significantly between samples, ranging from 349 to 1225.932 (Additional file 1: Fig. S2a). The AR sample had the largest Chao index, indicating that its bacterial species abundance was the highest, while BR had the lowest Chao index and the lowest bacterial species abundance. The Shannon indices of bacteria is between 4.579 and 7.523 and the Simpson indices is between 0.819 and 0.977 (Additional file 3: Table S3). The Chao indices of fungal reads ranged from 359.36 to 785.89 (Additional file 1: Fig. S2b). The ZW sample had the largest Chao index, indicating that its fungal species abundance was the highest, while M4C and H4C had the lowest Chao index and the lowest fungal species abundance. The Shannon indices of bacteria is between 3.107 and 5.055 and the Simpson indices is between 0.652 and 0.735 (Additional file 5: Table S4).

### Unique and shared ASVs analysis

**Fig. 1:**
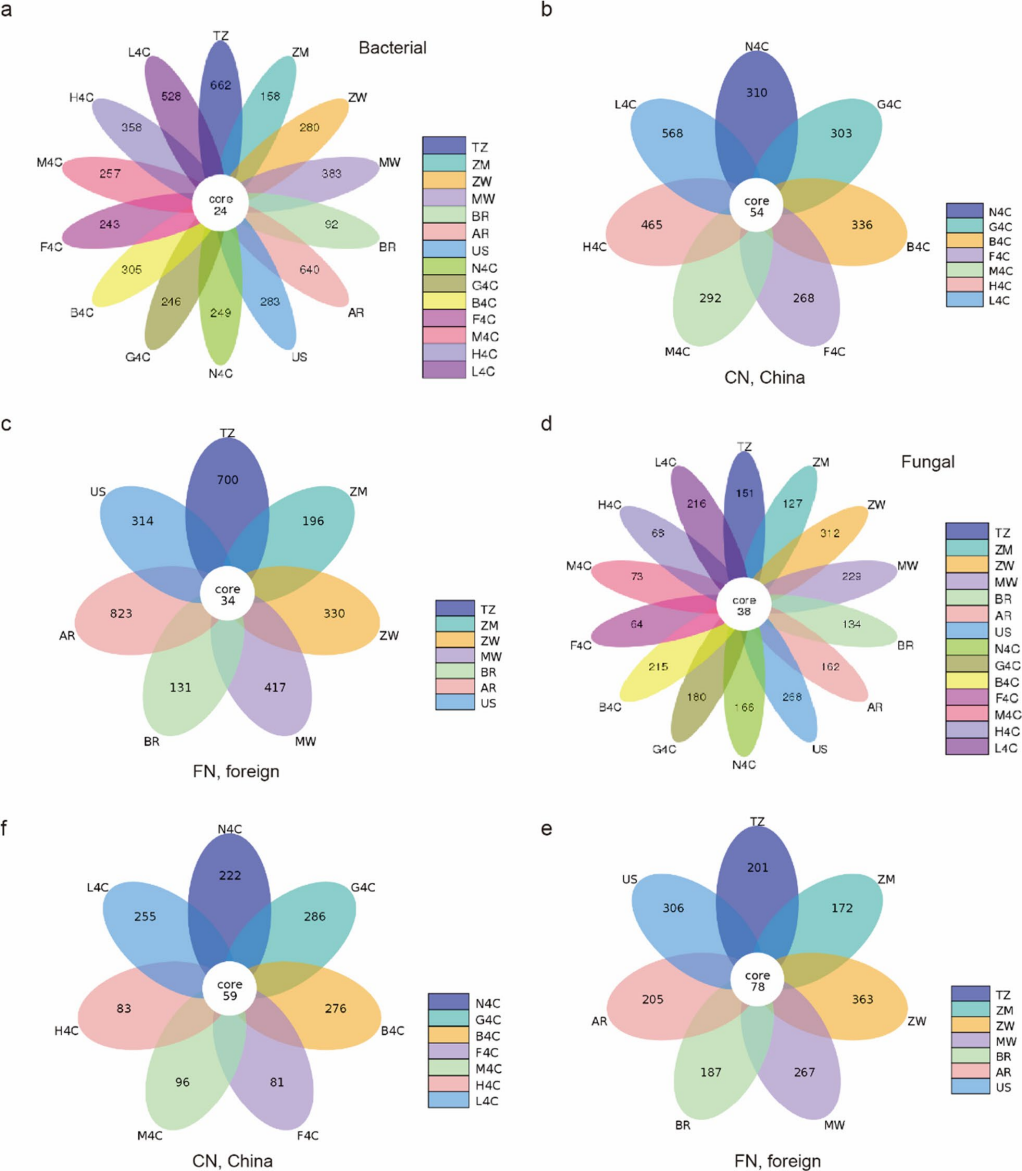
Petal plot of differences in bacterial **a**–**c** and fungal **d**–**f** diversity in 14 tobacco leaf samples. Each oval in the figure represents a sample, the number in the internal circle represents the number of ASVs common and the overlapping part of the circle represents numbers without overlap represent of samples. (e.g., the total number of TZ total is 662 unique plus 24 shared; Domestic and foreign samples were combined and grouped into CN and FN for analysis, FN, foreign; CN, China)

Species annotation was made for each representative of ASV obtained, resulting in 856 genera of bacteria in 29 phyla and fungi of 460 genera in 9 phyla. In terms of bacteria, a comparative analysis of bacterial ASVs on the surface of tobacco leaves after threshing and redrying found that 24 of them were identical (Fig. 1a). In domestic tobacco leaves, 54 ASVs were shared ASVs (Fig. 1b), and 34 ASVs were shared bacteria ASVs from the foreign tobacco leaves (Fig. 1c). Among the 7 tobacco leaf samples from abroad, the largest number of unique ASVs was Tanzania (TZ) tobacco with 662, followed by Argentina (AR) and Malawi (MW) tobacco leaves, with 640 and 383 respectively, and the number of unique ASVs in other countries was between 92 and 283. Among the 7 tobacco leaf samples in China, except for Lufeng tobacco in Yunnan (L4C), which had a high number of ASVs (528), the number of ASVs in the other six places was relatively low, between 243 and 358 (Fig. 1a). Further analysis of the differences in tobacco leaf bacterial community composition at home and abroad, the results of PCA analysis showed that except for Argentina (AR), Tanzania (TZ), Lufeng in Yunnan (L4C) and Huidong in Sichuan (H4C), the differences in the community structure of tobacco leaf bacteria were relatively small (Additional file 1: Fig. S3a). Similarly, according to the results of ASVs obtained by noise reduction, the common and unique fungal ASVs between tobacco leaf samples after threshing and redrying from different origins were analyzed. The results showed that 38 ASVs were common to all samples (Fig. 1d). In domestic tobacco leaves, 59 ASVs were shared ASVs (Fig. 1e), and 78 ASVs were shared fungal ASVs from the foreign tobacco leaves (Fig. 1f). In terms of unique ASVs, Zimbabwe (ZW) had the highest number of tobacco leaf samples abroad, with 312, followed by the United States (US) and Malawi (MW) with 268 and 229 respectively, and the number of unique ASVs in other countries was close, between 134 and 162. Among the domestic tobacco leaves, Lufeng in Yunnan (L4C) and Bozhou in Guizhou (B4C) had more unique ASVs, with 216 and 215 respectively, and the number of unique ASVs in the other 5 places was between 64 and 180. Overall, Zimbabwe (ZW), United States (US) and Malawi (MW) had more unique fungi (Fig. 1d). The results of PCA analysis showed that except for Zimbabwe (ZW) and United States (US), the differences in the community structure of tobacco leaf fungi were relatively small (Additional file 1: Fig. S3b).

### Bacterial community composition

**Fig. 2:**
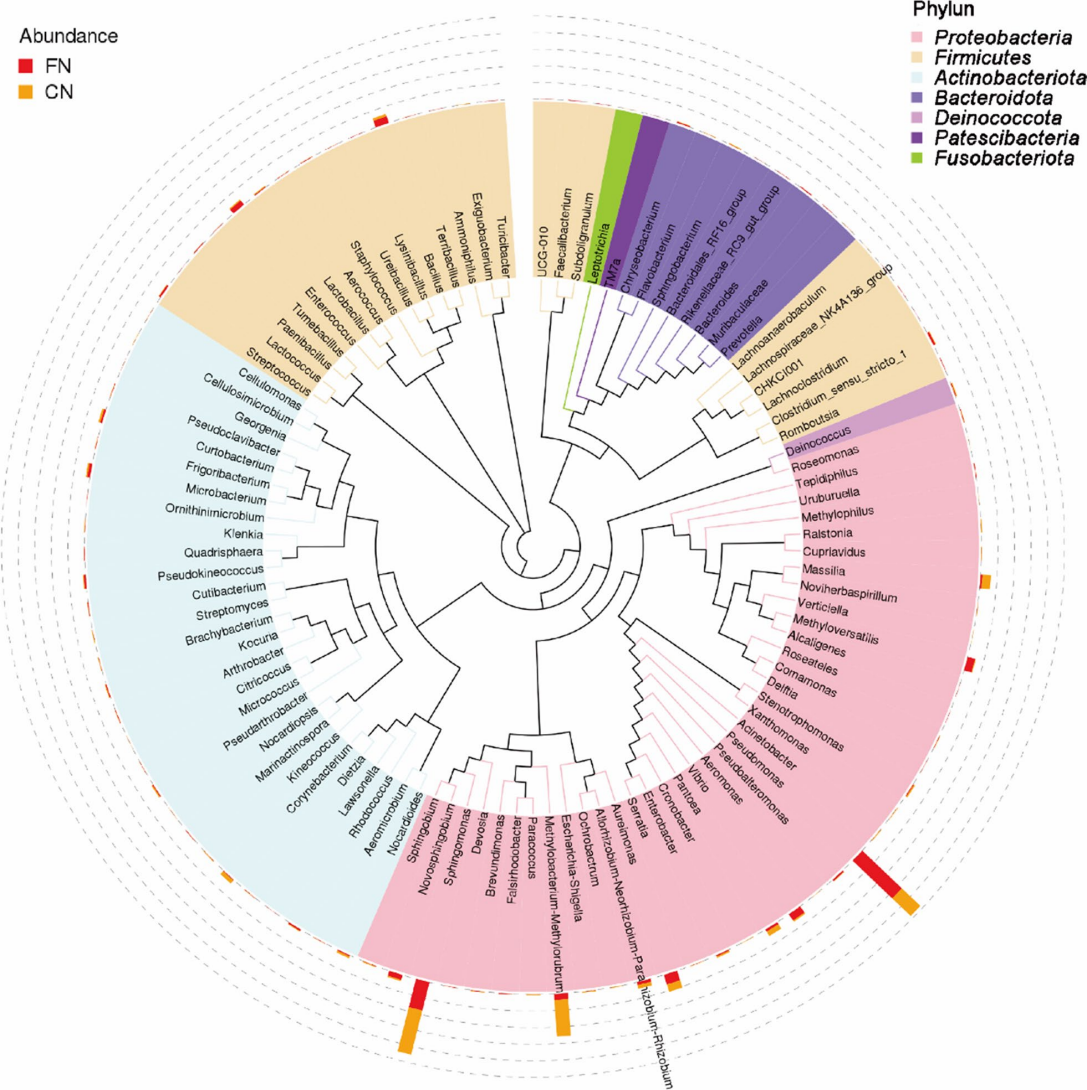
Phylogenetic tree of tobacco leaf bacteria at the genus level using the approximately-maximum-likelihood method. (Domestic and foreign samples were combined and grouped into CN (China) and FN (foreign) for analysis; CN samples were indicated in red in the figure and FN samples were indicated in orange in the figure)

**Fig. 3:**
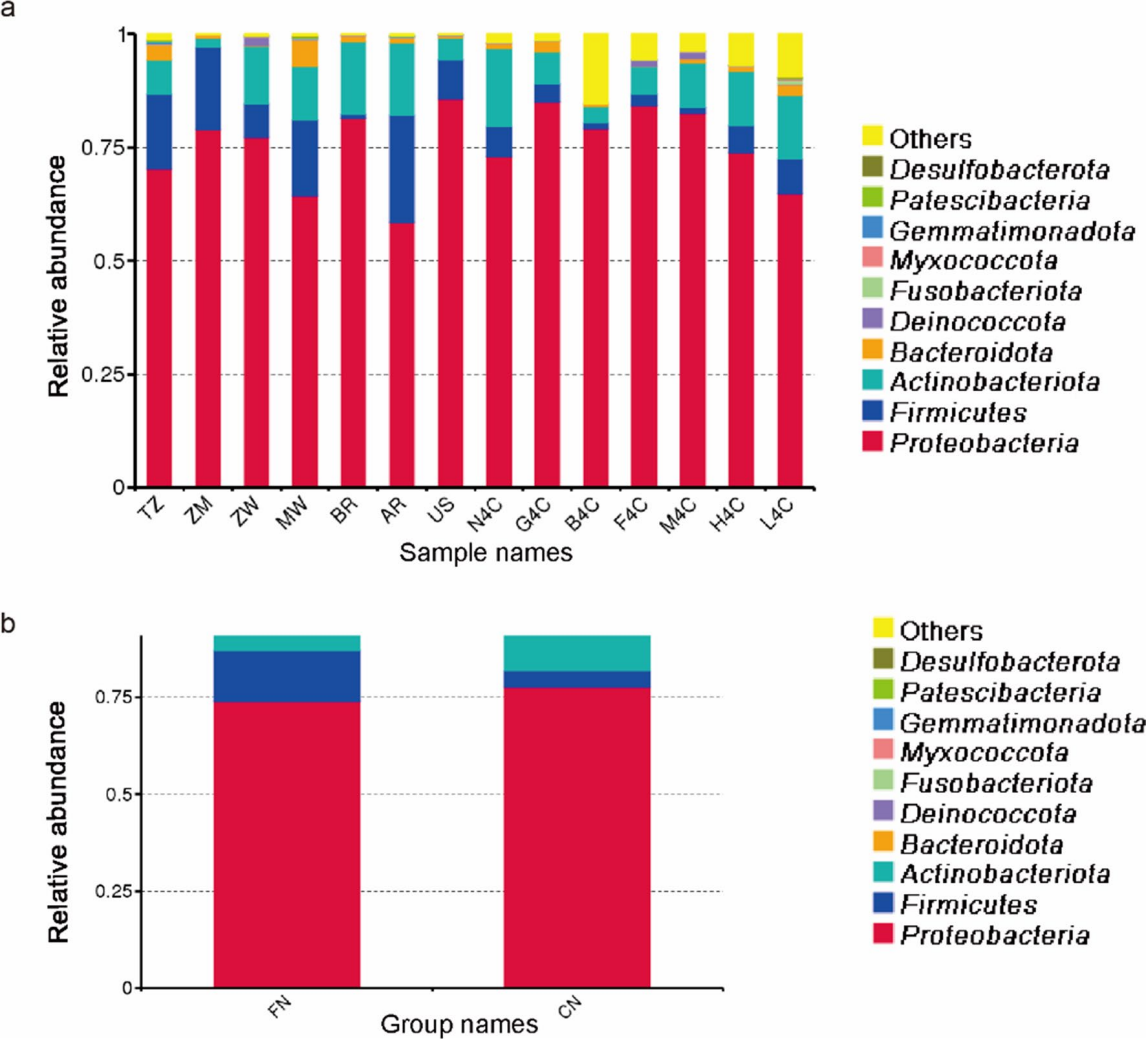
Bacterial communities of tobacco leaf at phyla level. The top10 bacterial phyla are indicated by different colors, and “others” represent the remaining members. **a** 14 samples were directly compared; **b** Comparison after grouping 14 samples into FN (foreign) and CN (China)

**Fig. 4:**
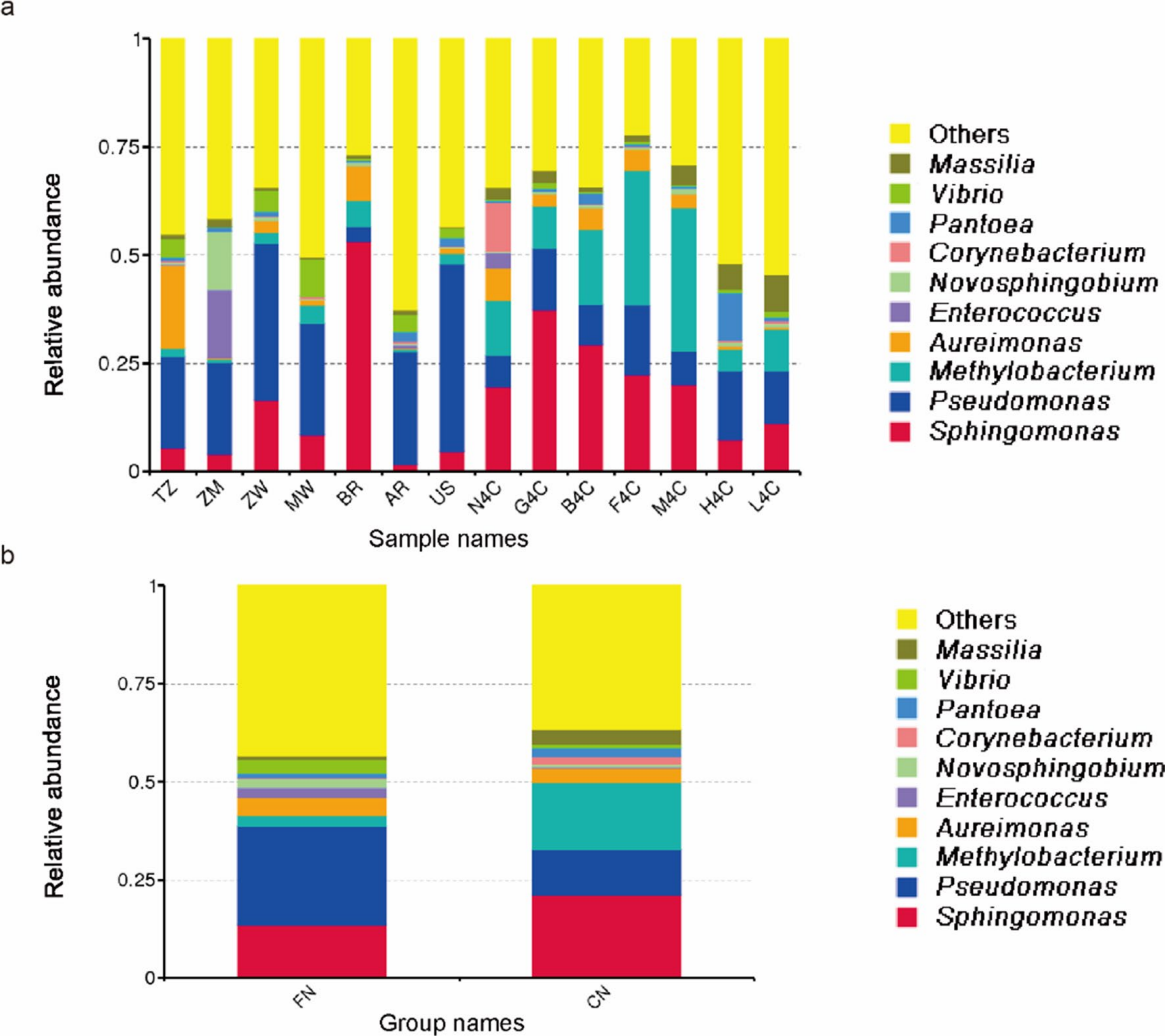
Bacterial communities on tobacco leaves at genus level after threshing and redrying. The top10 bacterial genera are indicated by different colors, and “others” represent the remaining members. **a** 14 samples were directly compared; **b** Comparison after grouping 14 samples into FN (foreign) and CN (China)

The bacterial ASVs identified in the 14 groups mainly belong to the phylum *Firmicutes*, *Proteobacteria*, *Actinomycetes*, and *Bacteroides*. Phylogenetic tree analysis of the representative sequences of the top 100 genera showed that *Proteobacteria* had the highest bacterial abundance and diversity, followed by *Firmicutes* and *Actinobacteriota*. *Deinococcota* and *Fusobacteria* had the fewest bacteria (Fig. 2). The bacteria with the highest abundance in the *Proteus* phylum are *Pseudomonas* and *Sphingomonas* bacteria, followed by bacteria of *Methylobacterium* and *Aureimonas*. The genus *Bacillus* in the phylum *Firmicutes* is the most abundant, followed by *Enterococcus*. At the taxonomic level of the phylum, the bacterial community structure of the top 10 in relative abundance was plotted, and the microflora with less than the top 10 abundance was classified as other (Fig. 3). Among them, the relative abundance of *Proteobacteria* in 14 samples was more than 50%. The relative abundance of *Firmicutes* in foreign tobacco samples was significantly higher than that of domestic tobacco leaves (Fig. 3b). Plot histograms of bacterial relative abundance was analyzed at the family level. The results showed that the top ten bacterial families in abundance in tobacco leave samples at home and abroad were *Sphingomonadaceae*, *Pseudomonadaceae*, *Beijerinckiaceae*, *Rhizobiaceae*, *Erwiniaceae*, *Enterococcaceae*, and *Corynebacteriaceae*, *Oxalobacteraceae*, *Enterobacteriaceae*, and *Comamonadaceae* (Additional file 1: Fig. S4). Except for Brazil, the relative abundance of *Pseudomonas* bacteria on the surface of other foreign tobacco leaves was relatively large, while the relative abundance of *Pseudomonas* in domestic tobacco leaves was relatively low (Additional file 1: Fig. S4a). The microbial communities on the surface of tobacco leaves at home and abroad were analyzed at the taxonomic level of this genus (Fig. 4). The results showed that there were certain differences in the bacterial community structure on the surface of tobacco leaves in different production areas. The relative abundance of *Microbacterium* and *Sphingomonas* in domestic tobacco leaves was significantly higher than that of foreign tobacco leaves (Fig. 4b). The relative abundance of *Sphingomonas* in foreign tobacco leaves was generally low, except for the relatively high relative abundance of tobacco leaves in Brazil (BR) and Zambia (ZM), 53% and 16%, respectively, and less than 10% of other exotic tobacco bacteria (Fig. 4a). The relative abundance of *Sphingomonas* in domestic tobacco leaves was relatively high, except for 7% in Sichuan Huidong tobacco leaves (H4C), the relative abundance of *Sphingomonas* in other domestic tobacco leaves was more than 10% (Fig. 4a). The relative abundance of *Microbacterium* in the composition of different tobacco leaf surface bacteria in China was between 5 and 31%, while the relative abundance of foreign tobacco leaf bacteria was low, between 0.7 and 6%. The relative abundance of *Pseudomonas* in foreign tobacco bacterial colonies was significantly higher than that of domestic tobacco leaves, accounting for 21–43% (except Brazil).

**Fig. 5:**
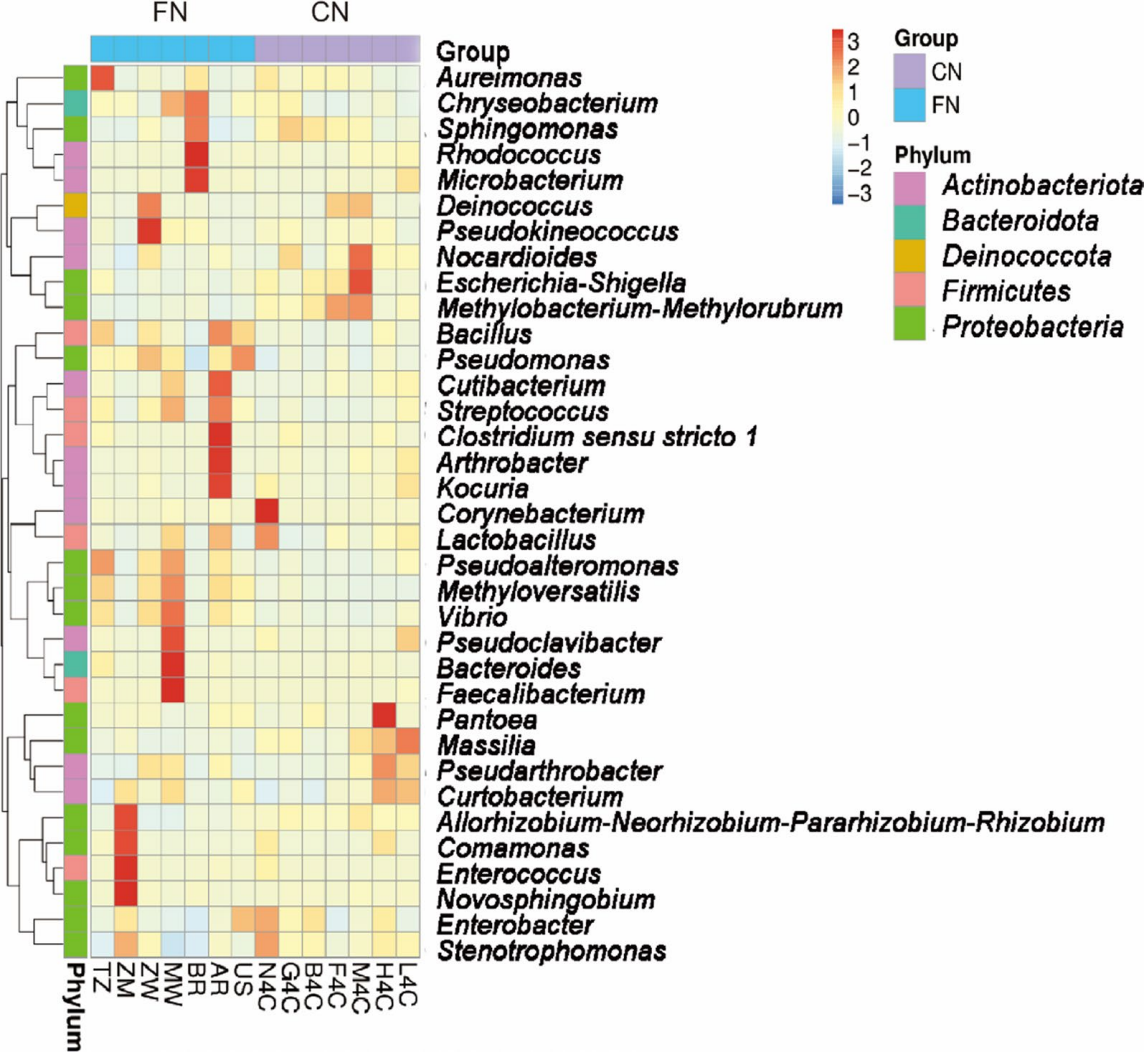
Heat map analyze the aggregation content of top 35 bacterial genera in each sample. Each column represents a sample, and each row designates fungal genera with relative abundance indicated by color bar. (FN, foreign; CN, China; CN samples were indicated in purple in the figure and FN samples were indicated in blue in the figure)

In addition, according to the species annotation and abundance information of all samples at the genus level, the top 35 genera in abundance were selected, and according to their abundance information in each sample, they were clustered by the two levels of bacterial taxa and samples, and heat maps were drawn to analyze the aggregation content of each bacterial species in each sample (Fig. 5). The results showed that the relative abundance of *Bacillus* in foreign tobacco leaves was generally higher than that of domestic tobacco leaves. The relative abundance of *Bacillus* was higher in Argentine, Tanzanian, and American tobacco leaves. The relative abundance of *Aureimonas* in Tanzania (TZ) tobacco leaves was significantly higher than that of other tobacco leaves. *Novosphingobium*, *Enterococcus*, *Comamonas* and *Allorhizobium* were the four dominant bacterial genera in Zambia (ZM) tobacco leaves. In the bacterial composition of tobacco leaf samples in Malawi (MW), the relative abundance of *Faecalibactrrium*, *Bacteroides*, and *Pseudoclavibacter* was significantly higher than that of other tobacco leaves. In the bacterial composition of tobacco leaves in Brazil (BR), *Microbacterium*, *Rhodococcus* and *Sphingomonas* accounted for a significantly higher proportion. Among the bacterial composition of tobacco leaves in Argentina (AR), *Kocuria*, *Arthrobacter* and *Clostridium* accounted for a relatively high proportion. Zimbabwe (ZW) has a large relative abundance of *Pseudokineococcus* and *Deinococcus*; The relative abundance of *Pseudomonas* in tobacco leaves in the United States (US) was relatively high (Fig. 5). The relative abundance of *Corynebacterium* in domestic N4C tobacco leaves was significantly higher than that of other tobacco leaves. The relative abundance of *Escherichia* and *Nocardioides* in M4C tobacco leaves was high. The relative abundance of *Pantoea* in Huidong tobacco was the highest, and the relative abundance of *Massilia* in Lufeng tobacco was high. These results suggested that geography was highly associated with the dominant bacteria present on the threshed and redried tobacco leaves.

### Fungal community composition

**Fig. 6:**
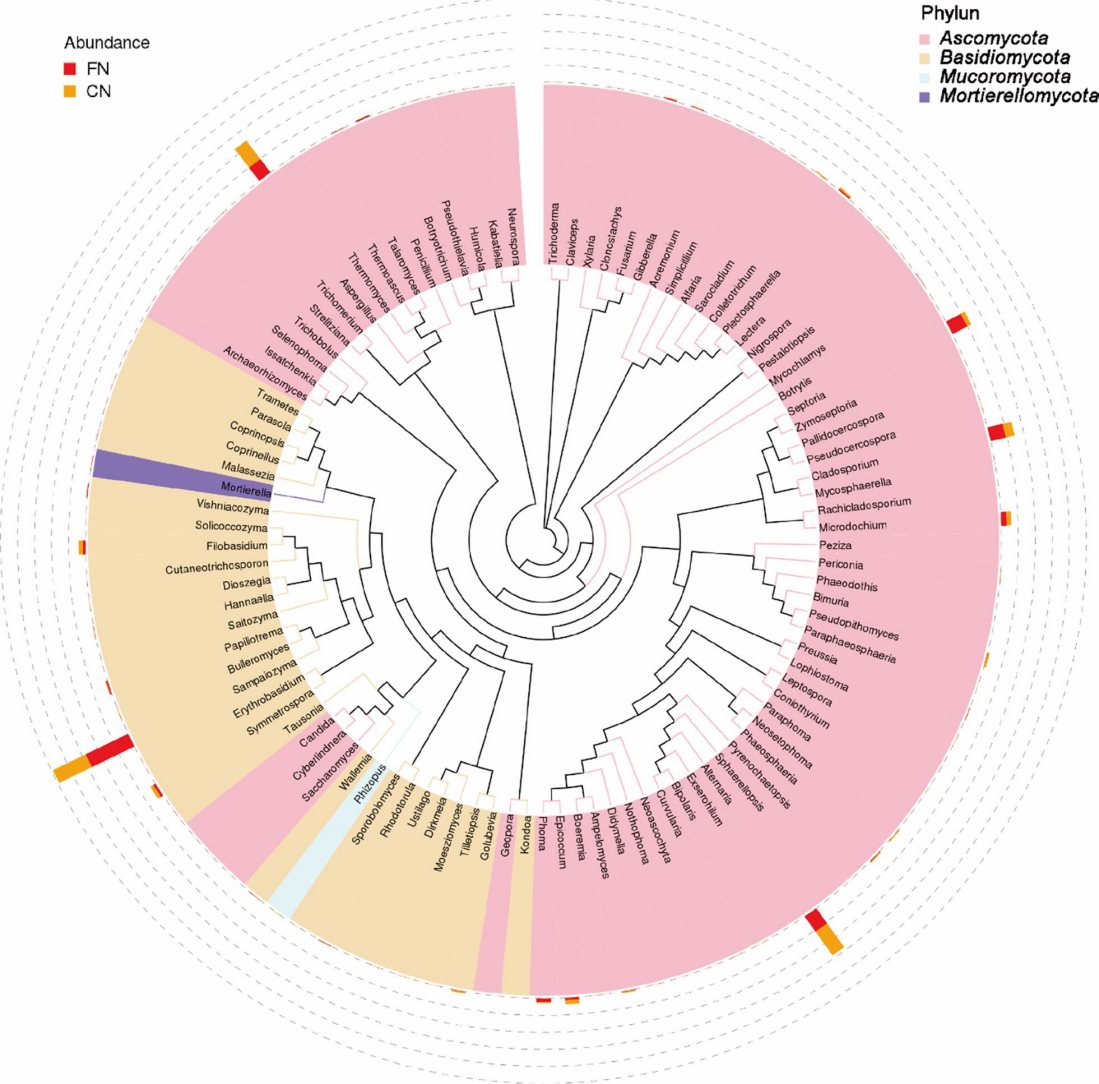
Phylogenetic tree of tobacco leaf fungi at the genus level using the approximately-maximum-likelihood method. (Domestic and foreign samples were combined and grouped into CN (China) and FN (foreign) for analysis; CN samples were indicated in red in the figure and FN samples were indicated in orange in the figure)

A total of 9 phyla and 459 genera of fungi were detected from 14 tobacco leaf samples. The dominant fungal phyla were *Ascomycota*, *Basidiomycota*, *Mucoromycota* and *Mortierellomycota*. Phylogenetic tree analysis of the representative sequences of the analyzed top 100 genera showed that *Ascomycota* had the highest fungal abundance and diversity, followed by *Basidiomycota* and *Mucoromycota* (Fig. 6). The *Mortierellomycota* has the fewest fungal species. The most abundant fungal genera in the *Ascomycota* were *Alternaria* and *Aspergillus*, followed by *Cladosporium* and *Septoria*. *Sampaiozyma* in the *Basidiomycota* had the highest fungal abundance, followed by *Filobasidium* and *Symmetrospora*. In the phylum *Mucoromycota* and the *Mortierellomycota*, there only had fungi of the *Rhizopus* and *Mortierella*, respectively. Plot histograms of fungi relative abundance was analyzed at the family level (Additional file 1: Fig. S5). The results showed that the top ten fungi families in abundance in 14 samples at home and abroad were *Mycosphaerellaceae*, *Chrysozymaceae*, *Didymellaceae*, *Pleosporaceae*, *Aspergillaceae*, *Cladosporiaceae*, *Microdochiaceae*, *Filobasidiaceae*, *Symmetrosporaceae* and *Golubeviaceae* (Additional file 1: Fig. S5). The relative abundance of *Mycosphaerellaceae*, *Chrysozymaceae* and *Symmetrosporaceae* in foreign tobacco fungi was significantly higher than that in domestic tobacco leaves, while the relative abundance of *Didymellaceae*, *Aspergillaceae* and *Pleosporaceae* in domestic tobacco fungi was higher than that of foreign tobacco leaves (Additional file 1: Fig. S5b). Zambia (ZM) had the highest relative abundance of *Mycosphaerellaceae*, about 58%. The relative abundance of *Mycosphaerellaceae* in other tobacco leaves was less than 10%. In addition, *Chrysozymaceae* had high relative abundance in Tanzania (TZ), Zimbabwe (ZW), Malawi (MW), Brazil (BR) and domestic N4C, G4C, B4C and F4C samples.

**Fig. 7:**
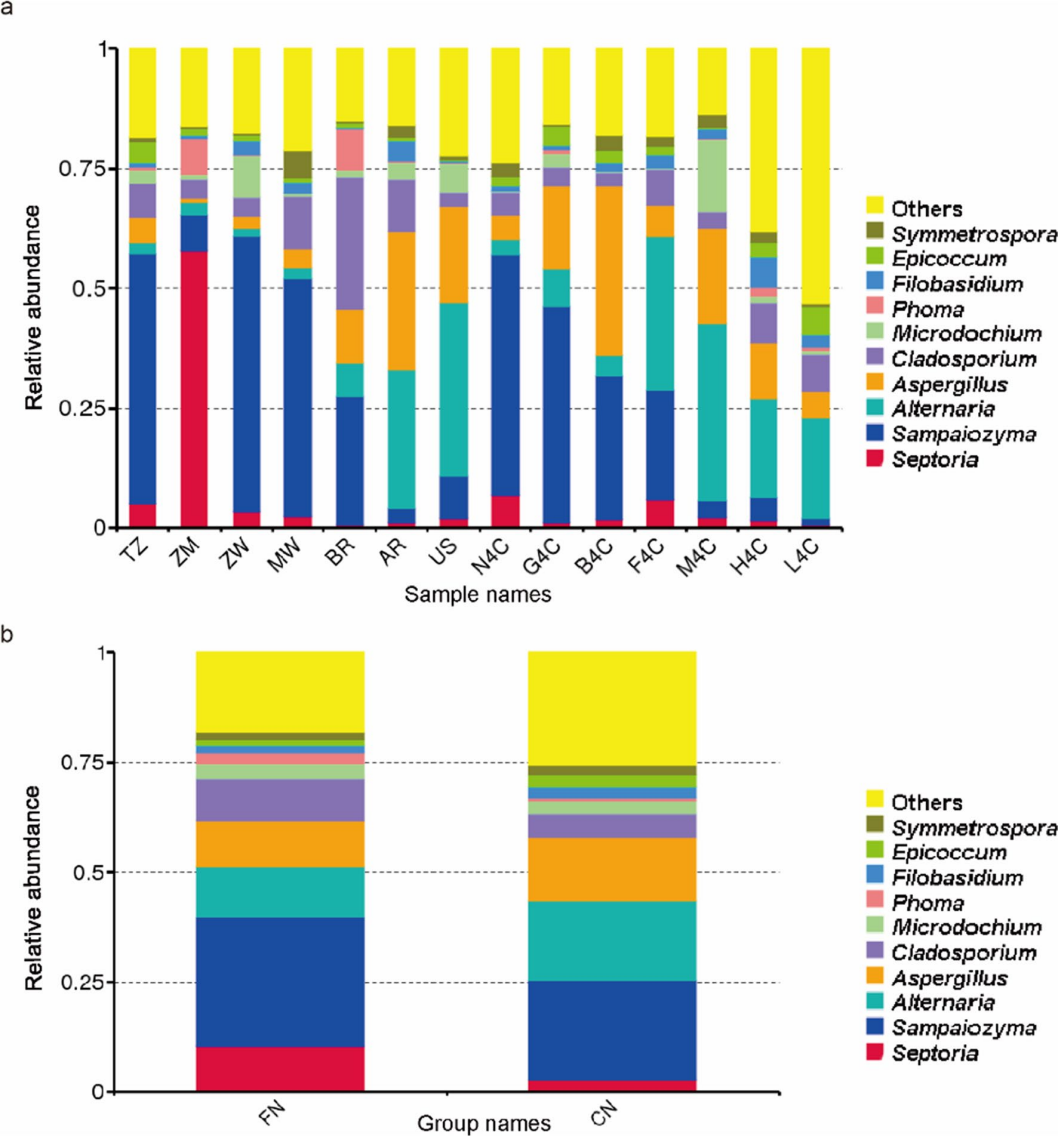
Fungi communities on tobacco leaves at genus level after threshing and redrying. The top10 fungi genera are indicated by different colors, and “others” represent the remaining members. **a** 14 samples were directly compared; **b** Comparison after grouping 14 samples into FN (foreign) and CN (China)

The composition of the fungal flora on the surface of tobacco leaves at home and abroad was analyzed at the taxonomic level of the genus (Fig. 7). The results showed that the relative abundance of *Aspergillus* and *Alternaria* in domestic tobacco leaves was significantly higher than that in foreign tobacco leaves. The relative abundance of *Septoria*, *Sampaiozyma*, *Cladosporium* and *Phoma* in foreign tobacco leaves was significantly higher than that of domestic tobacco leaves. In Zambian (ZM) tobacco leaves, *Septoria* was the most dominant genus, its relative abundance is as high as 57%, but the relative abundance of *Septoria* in other tobacco leaves fungi both domestic and foreign was less than 10%. The relative abundance of *Phoma* in Brazil (BR) and Zambia (ZM) was 53% and 16%, respectively, and less than 2% of other tobacco fungi. *Cladosporium* had the highest relative abundance in Brazil (BR) tobacco leaves at about 27%, followed by Malawi (MW) and Argentina (AR) with 11% and 10.8%, respectively, while the relative abundance of *Cladosporium* in other tobacco leaves was less than 10%.

**Fig. 8:**
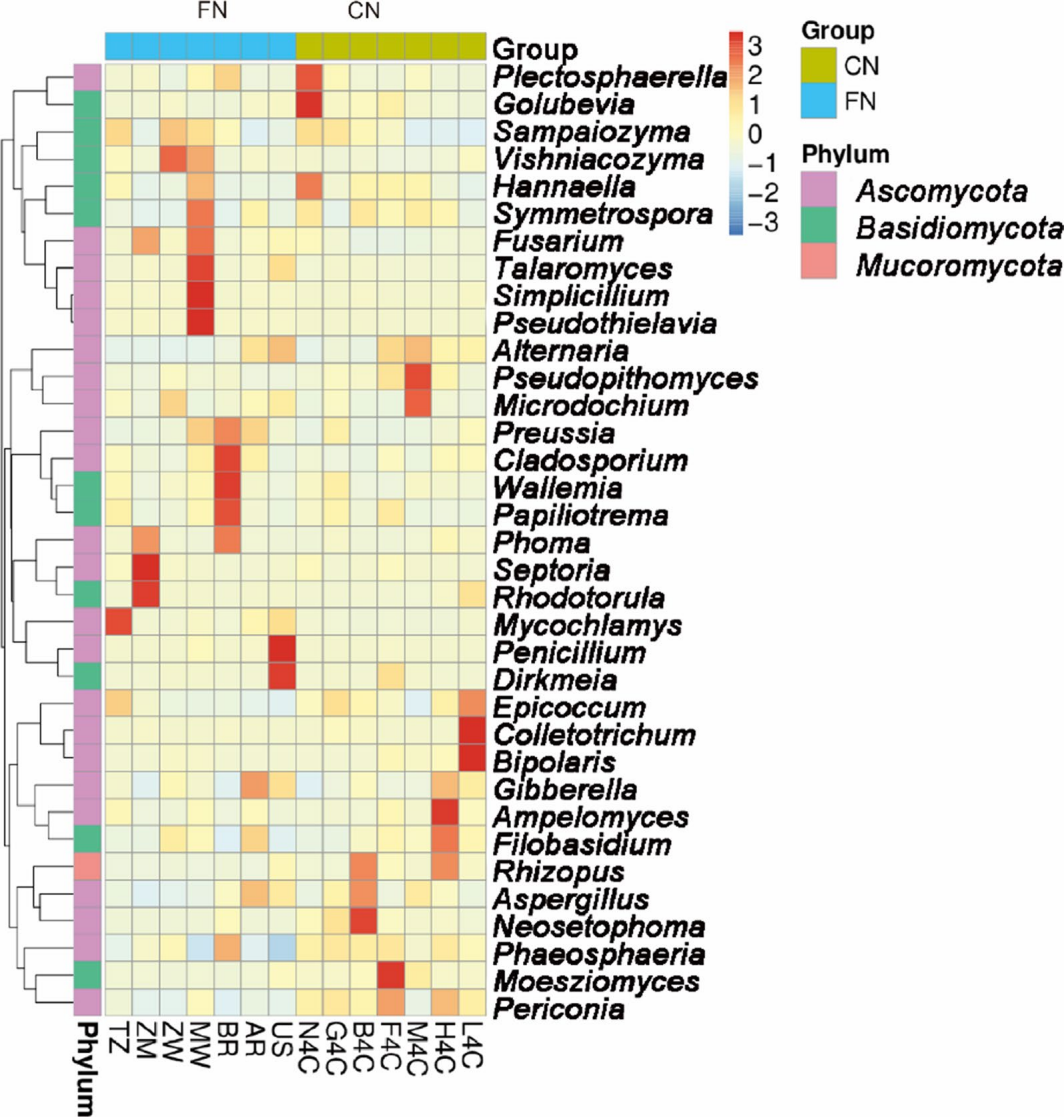
Heat map analyze the aggregation content of top 35 fungi genera in each sample. Each column represents a sample, and each row designates fungal genera with relative abundance indicated by color bar. (FN, foreign; CN, China; CN samples were indicated in green in the figure and FN samples were indicated in blue in the figure)

The top 35 genera in abundance were selected, clustered at the species and sample levels, heat maps were drawn, and the aggregation of each fungal species in each sample was analyzed (Fig. 8). The relative abundance of *Mycochlamys*, *Sampaiozyma*, and *Epicoccum* in Tanzania (TZ) was high. The relative abundance of *Phoma*, *Septoria*, *Rhodotorula*, *Sampaiozyma* and *Fusarium* in Zambia (ZM) was significantly higher than that of other tobacco leaves. Zimbabwe (ZW) had a relatively high abundance of *Vishniacozyma* and *Sampaiozyma*. Malawi (MW) tobacco leaves had more dominant fungi, such as *Pseudothielavia*, *Simplicillium*, *Talaromyces*, *Sampaiozyma*, *Fusarium*, *Hannaella* and *Vishniacozyma*. Brazil (BR) tobacco leaves had a significantly higher proportion of *Cladosporium*, *Wallemia*, *Papiliotrema*, *Phoma* and *Preussia*. In Argentine (AR) tobacco leaves, *Filobasidium* and *Aspergillus* were relatively abundant. *Penicillium* and *Dirkmeia* were the the most dominant genera in United States tobacco leaves. In domestic tobacco leaves, the dominant genera in Fujian Ninghua (N4C) fungi were *Golubevia*, *Plectosphaerella* and *Hannaella*. The proportions of *Neosetophoma*, *Aspergillus* and *Rhizopus* were significantly higher in Bozhou, Guizhou tobacco leaves. The relative abundance of *Moesziomyces* and *Periconia* in Feixian, Shandong (F4C) was significantly higher than that of other tobacco leaves. The proportion of *Pseudopithomyces*, *Microdochium* and *Alternaria* in Yingchi, Henan was relatively high. The dominant fungal genera in Huidong, Sichuan (H4C) were *Ampelomyces*, *Filobasidium*, *Rhizopus* and *Gibberella*. The relative abundance of *Colletotrichum*, *Bipolaris* and *Epicoccum* in Lufeng (L4C), Yunnan was higher than that of other tobacco leaves.

## Discussion

In this study, the Illumina HiSeq sequencing based on 16S rRNA and ITS1 gene was used to investigate the microbial community composition and diversity of redried tobacco leaves in China and abroad. The bacteria in tobacco leaves after redrying were mainly composed of *Proteobacteria*, *Firmicutes*, *Actinobacteria*, and *Bacteroidetes*. Among them, *Proteobacteria* was the most prominent phylum, with relative abundances of more than 50%. *Pseudomonas*, bacteria of the *Proteobacteria* phylum, had a relative abundance of 25% in foreign tobacco leaves, compared to only 11% in domestic tobacco leaves (Fig. 3). In previous studies, *Pseudomonas* spp. has been shown to be a dominant genera bacterial component in flue-cured tobacco (Huang et al. 2010) and to play an important role in nicotine degradation in tobacco leaves (Wang et al. 2004; Zhong et al. 2010). The relative abundance of *Firmicutes* in foreign tobacco samples was significantly higher than that of domestic tobacco leaves. The relative abundance of *Firmicutes* was 13% in foreign tobacco leaves and only 4% in domestic tobacco leaves. *Bacillus* belongs to *Firmicutes* was another dominant genus in the tobacco leaf bacteria. *Bacillus* is one of the dominant genera in aged tobacco and plays an important role in improving leaf quality during tobacco aging (Ye et al. 2017; Dai et al. 2020; Huang et al. 2022; Wu et al. 2023). Studies have found that after inoculating *Bacillus* onto the surface of tobacco, with a decrease in total carbohydrates and a decrease in reducing sugars in tobacco leaves, a pleasant aroma was produced (English et al. 1967). In addition, Maldonado-Robledo et al. (2003) found that *Bacillus* can produce small aromatic substances by breaking down large molecules such as carotene. The relative abundance of core microorganisms such as *Bacillus* was an important factor affecting the quality of tobacco leaves, which can be used to improve tobacco quality.

This study found further that the bacteria in redried tobacco leaves mainly included *Sphingomonadales*, *Pseudomonadales*, *Methylobacterium*, *Aureimonas*, *Enterococcus*, *Novosphingobium*, *Corynebacterium*, and *Pantoea* (Fig. 4). These genera have been reported to play an important role in nicotine degradation and the formation of representative flavor compounds (Wang et al. 2018b; Zhang et al. 2019; Ye et al. 2021). The relative abundance of *Pseudomonas* and *Sphingomycetes* varies significantly during aging, suggesting that they play an important role in aging progress (Zhou et al. 2020). Several studies had also confirmed that the degradation of nicotine is related to *Pseudomonas* (Law et al. 2016). *Methylobacterium* plays a role in degrading organic acids, sugars, formaldehyde, formate, and methanol present in tobacco (Madhaiyan and Poonguzhali 2014). *Sphingomonas* sp. isolated from tobacco leaves can degrade chlorogenic acid into to caffeic acid, shikimic acid, and 3,4-dihydroxybenzoic acid, involved in the tobacco leaf flavor formation process (Ma et al. 2016).

In terms of fungi, in redried tobacco leaves *Ascomycetes* and *Basidiomycetes* were the main fungal phylum, and *Sampaiozyma*, *Aspergillus* and *Alternaria* phyla were the dominant genera (Fig. 7). This finding did not agree with some earlier reports. Zeng et al. (2014) and Chen et al. (2020) found that *Rhizopus* was the dominant culturable fungus, followed by *Aspergillus* sp during barn rot in flue-cured tobacco. In addition, Zhang et al. (2019) found that *Cladosporium* and *Neophaeosphaeria* were the dominant genera in flue-cured tobacco leaves. We found that the relative abundance of *Aspergillus* and *Alternaria* in domestic tobacco leaves was significantly higher than that in foreign tobacco leaves. The relative abundance of *Sampaiozyma* in foreign tobacco leaves is significantly higher than that of domestic tobacco leaves. *Aspergillus oryzae* 112822 has been shown to degrade nicotine to 2,3-dihydroxypyridine. This process was achieved by the intermediates nornicotine, myosmine, *N*-methylnicotinamide and 2-hydroxy-*N*-methylnicotinamide (Meng et al. 2010). These results suggesting that fungi such as *Aspergillus* play a role in tobacco leaves aging. This study also found that the fungi species in tobacco leaves at home and abroad were different (Fig. 7). Zimbabwe and the United States tobacco leaves had more unique fungal species. The relative abundance of *Sampaiozyma*, *Vishniacozyma* and *Microdochium* was relatively high in Zimbabwean tobacco leaves. In American tobacco leaves, *Penicillium*, *Dirkmeia*, *Gibberella*, etc. were the dominant fungal genera. *Sampaiozyma* was relatively abundant in Tanzania, Zimbabwe, Malawi and Brazil, and *Vishniacozyma* was high in Tanzania, Zimbabwe and Malawi. *Preussia* and *Cladosporium* were more abundant in Malawi, Brazil and Argentina than in other tobacco leaves. However, it is important to note that most of these detected fungi (*Penicillium*, *Cladosporium*, *Aspergillus* and *Alternaria*) cause mold, which is detrimental to tobacco storage (Welty and Lucas 1968; Welty et al. 1968). Therefore, how to effectively use these fungi to reduce the content of harmful substances such as nicotine tobacco leaves without affecting tobacco storage needs further research.

## Supplementary Information

**Additional file 1: Figure S1.** The rough location of the tobacco leaf sample marked on the world map. The pentagram indicates the approximate location of domestic tobacco samples, and the dots indicates the approximate location of foreign tobacco samples. **Figure S2.** Diversity and composition of bacterial and fungal communities in tobacco leaves after threshing and redrying. a) Chao richness values of bacterial communities b) Chao richness values of fungal communities. **Figure S3.** PCA analysis of bacterial (a) and fungal (b) communities in 14 tobacco leaf samples at ASV level. (FN, foreign; CN, China; CN samples were indicated in blue in the figure and FN samples were indicated in red in the figure). **Figure S4.** Bacterial communities of tobacco leaf at family level. The top10bacterial families are indicated by different colors, and “others” represent the remaining members. (a) 14 samples were directly compared; (b) Comparison after grouping 14 samples into FN (foreign) and CN (China). **Figure S5.** Fungi communities of tobacco leaf at family level. The top10 fungi families are indicated by different colors, and “others” represent the remaining members. (a) 14 samples were directly compared; (b) Comparison after grouping 14 samples into FN (foreign) and CN (China).

**Additional file 2: Table S1.** The 16S rRNA sequencing data of tobacco leaf samples after threshing and redrying.

**Additional file 3: Table S2.** The ITS1 sequencing data of tobacco leaf samples after threshing and redrying.

**Additional file 4: Table S3.** ASV of 16S rRNA sequences analyzed.

**Additional file 5: Table S4.** ASV of ITS1 sequences analyzed.

## Data Availability

All data generated or analyzed during this study are available from the corresponding author on reasonable request.

## References

[CR1] Callahan BJ, McMurdie PJ, Holmes SP (2017). Exact sequence variants should replace operational taxonomic units in marker-gene data analysis. ISME J.

[CR2] Caporaso JG, Kuczynski J, Stombaugh J, Bittinger K, Bushman FD, Costello EK, Fierer N, Peña AG, Goodrich JK, Gordon JI, Huttley GA, Kelley ST, Knights D, Koenig JE, Ley RE, Lozupone CA, McDonald D, Muegge BD, Pirrung M, Reeder J, Sevinsky JR, Turnbaugh PJ, Walters WA, Widmann J, Yatsunenko T, Zaneveld J, Knight R (2010). QIIME allows analysis of high-throughput community sequencing data. Nat Methods.

[CR3] Chen Q-L, Cai L, Wang H-C, Cai L-T, Goodwin P, Ma J, Wang F, Li Z (2020). Fungal composition and diversity of the tobacco leaf phyllosphere during curing of leaves. Front Microbiol.

[CR4] Dai J, Dong A, Xiong G, Liu Y, Hossain MdS, Liu S, Gao N, Li S, Wang J, Qiu D (2020). Production of highly active extracellular amylase and cellulase from *Bacillus subtilis* ZIM3 and a recombinant strain with a potential application in tobacco fermentation. Front Microbiol.

[CR5] Di Giacomo M, Paolino M, Silvestro D, Vigliotta G, Imperi F, Visca P, Alifano P, Parente D (2007). Microbial community structure and dynamics of dark fire-cured tobacco fermentation. Appl Environ Microbiol.

[CR6] English CF, Bell EJ, Berger AJ (1967). Isolation of thermophiles from broadleaf tobacco and effect of pure culture inoculation on cigar aroma and mildness. Appl Microbiol.

[CR7] Huang J, Yang J, Duan Y, Gu W, Gong X, Zhe W, Su C, Zhang K-Q (2010). Bacterial diversities on unaged and aging flue-cured tobacco leaves estimated by 16S rRNA sequence analysis. Appl Microbiol Biotechnol.

[CR8] Huang S, Liu D, Chen M, Xi G, Yang P, Jia C, Mao D (2022). Effects of *Bacillus subtilis* subsp. on the microbial community and aroma components of flue-cured tobacco leaves based on metagenome analysis. Arch Microbiol.

[CR9] Hugenholtz P, Goebel BM, Pace NR (1998). Impact of culture-independent studies on the eemerging phylogenetic view of bacterial diversity. J Bacteriol.

[CR10] Jo J, Oh J, Park C (2020). Microbial community analysis using high-throughput sequencing technology: a beginner’s guide for microbiologists. J Microbiol Seoul Korea.

[CR11] Kõljalg U, Nilsson RH, Abarenkov K, Tedersoo L, Taylor AFS, Bahram M, Bates ST, Bruns TD, Bengtsson-Palme J, Callaghan TM, Douglas B, Drenkhan T, Eberhardt U, Dueñas M, Grebenc T, Griffith GW, Hartmann M, Kirk PM, Kohout P, Larsson E, Lindahl BD, Lücking R, Martín MP, Matheny PB, Nguyen NH, Niskanen T, Oja J, Peay KG, Peintner U, Peterson M, Põldmaa K, Saag L, Saar I, Schüßler A, Scott JA, Senés C, Smith ME, Suija A, Taylor DL, Telleria MT, Weiss M, Larsson K-H (2013). Towards a unified paradigm for sequence-based identification of fungi. Mol Ecol.

[CR12] Law AD, Fisher C, Jack A, Moe LA (2016). Tobacco, microbes, and carcinogens: correlation between tobacco cure conditions, tobacco-specific nitrosamine content, and cured leaf microbial community. Microb Ecol.

[CR13] Li M, Shao D, Zhou J, Gu J, Qin J, Chen W, Wei W (2020). Signatures within esophageal microbiota with progression of esophageal squamous cell carcinoma. Chin J Cancer Res.

[CR14] Liu J, Ma G, Chen T, Hou Y, Yang S, Zhang K-Q, Yang J (2015). Nicotine-degrading microorganisms and their potential applications. Appl Microbiol Biotechnol.

[CR15] Ma Y, Wang X, Nie X, Zhang Z, Yang Z, Nie C, Tang H (2016). Microbial degradation of chlorogenic acid by a *Sphingomonas* sp. strain. Appl Biochem Biotechnol.

[CR16] Madhaiyan M, Poonguzhali S (2014). *Methylobacterium pseudosasae* sp. nov., a pink-pigmented, facultatively methylotrophic bacterium isolated from the bamboo phyllosphere. Antonie Van Leeuwenhoek.

[CR17] Magoč T, Salzberg SL (2011). FLASH: fast length adjustment of short reads to improve genome assemblies. Bioinformatics.

[CR18] Maldonado-Robledo G, Rodriguez-Bustamante E, Sanchez-Contreras A, Rodriguez-Sanoja R, Sanchez S (2003). Production of tobacco aroma from lutein. Specific role of the microorganisms involved in the process. Appl Microbiol Biotechnol.

[CR19] Meng XJ, Lu LL, Gu GF, Xiao M (2010). A novel pathway for nicotine degradation by *Aspergillus oryzae* 112822 isolated from tobacco leaves. Res Microbiol.

[CR20] Nilsson RH, Anslan S, Bahram M, Wurzbacher C, Baldrian P, Tedersoo L (2019). Mycobiome diversity: high-throughput sequencing and identification of fungi. Nat Rev Microbiol.

[CR21] Rognes T, Flouri T, Nichols B, Quince C, Mahé F (2016). VSEARCH: a versatile open source tool for metagenomics. PeerJ.

[CR22] Sajid M, Srivastava S, Yadav RK, Joshi L, Bharadwaj M (2023). Fungal community composition and function associated with loose smokeless tobacco products. Curr Microbiol.

[CR23] Vigliotta G, Di Giacomo M, Carata E, Massardo DR, Tredici SM, Silvestro D, Paolino M, Pontieri P, Del Giudice L, Parente D, Alifano P (2007). Nitrite metabolism in debaryomyces hansenii TOB-Y7, a yeast strain involved in tobacco fermentation. Appl Microbiol Biotechnol.

[CR24] Wang SN, Xu P, Tang HZ, Meng J, Liu XL, Huang J, Chen H, Du Y, Blankespoor HD (2004). Biodegradation and detoxification of nicotine in tobacco solid waste by a *Pseudomonas* sp. Biotechnol Lett.

[CR25] Wang F, Men X, Zhang G, Liang K, Xin Y, Wang J, Li A, Zhang H, Liu H, Wu L (2018). Assessment of 16S rRNA gene primers for studying bacterial community structure and function of aging flue-cured tobaccos. AMB Express.

[CR26] Wang F, Zhao H, Xiang H, Wu L, Men X, Qi C, Chen G, Zhang H, Wang Y, Xian M (2018). Species diversity and functional prediction of surface bacterial communities on aging flue-cured tobaccos. Curr Microbiol.

[CR27] Welty RE, Lucas GB (1968). Fungi isolated from damaged flue-cured tobacco1. Appl Microbiol.

[CR28] Welty RE, Lucas GB, Fletcher JT, Yang H (1968). Fungi isolated from tobacco leaves and brown-spot lesions before and after flue-curing. Appl Microbiol.

[CR29] Wu X, Cai W, Zhu P, Peng Z, Zheng T, Li D, Li J, Zhou G, Zhang J, Du G (2023). Function-driven design of *Bacillus kochii* and *Filobasidium magnum* co-culture to improve quality of flue-cured tobacco. Front Microbiol.

[CR30] Ye J, Yan J, Zhang Z, Yang Z, Liu X, Zhou H, Wang G, Hao H, Ma K, Ma Y, Mao D, Yang X (2017). The effects of threshing and redrying on bacterial communities that inhabit the surface of tobacco leaves. Appl Microbiol Biotechnol.

[CR31] Ye J, Ding Y, Qi X, Xu J, Yang X, Zhang Z (2021). Geographic and position-based variations in phyllospheric bacterial communities present on flue-cured tobacco. Appl Microbiol Biotechnol.

[CR32] Zeng T, Gu G, Zhang S (2014). Identification of tobacco mildew pathogen which causes leaf rot during flue-curing. Acta Tabacaria Sin.

[CR33] Zhang Q, Geng Z, Li D, Ding Z (2019). Characterization and discrimination of microbial community and co-occurrence patterns in fresh and strong flavor style flue-cured tobacco leaves. Microbiologyopen.

[CR34] Zhao M, Wang B, Li F, Qiu L, Li F, Wang S, Cui J (2007). Analysis of bacterial communities on aging flue-cured tobacco leaves by 16S rDNA PCR-DGGE technology. Appl Microbiol Biotechnol.

[CR35] Zhong W, Zhu C, Shu M, Sun K, Zhao L, Wang C, Ye Z, Chen J (2010). Degradation of nicotine in tobacco waste extract by newly isolated *Pseudomonas* sp. ZUTSKD. Bioresour Technol.

[CR36] Zhou J, Yu L, Zhang J, Zhang X, Xue Y, Liu J, Zou X (2020). Characterization of the core microbiome in tobacco leaves during aging. Microbiologyopen.

[CR37] Zhou J, Cheng Y, Yu L, Zhang J, Zou X (2022). Characteristics of fungal communities and the sources of mold contamination in mildewed tobacco leaves stored under different climatic conditions. Appl Microbiol Biotechnol.

