# Supplementary material for: Analysis of differences in tobacco leaf microbial communities after redrying in Chinese provinces and from abroad
Source: AMB Express. 2023 Aug 1;13:80. doi: 10.1186/s13568-023-01580-5 (PMC10393934; doi:10.1186/s13568-023-01580-5)
Supplement: Supplementary file 1 — Additional file 1: Figure S1. The rough location of the tobacco leaf sample marked on the world map. The pentagram indicates the approximate location of domestic tobacco samples, and the dots indicates the approximate location of foreign tobacco samples. Figure S2. Diversity and composition of bacterial and fungal communities in tobacco leaves after threshing and redrying. a) Chao richness values of bacterial communities b) Chao richness values of fungal communities. Figure S3. PCA analysis of bacterial (a) and fungal (b) communities in 14 tobacco leaf samples at ASV level. (FN, foreign; CN, China; CN samples were indicated in blue in the figure and FN samples were indicated in red in the figure). Figure S4. Bacterial communities of tobacco leaf at family level. The top10bacterial families are indicated by different colors, and “others” represent the remaining members. (a) 14 samples were directly compared; (b) Comparison after grouping 14 samples into FN (foreign) and CN (China). Figure S5. Fungi communities of tobacco leaf at family level. The top10 fungi families are indicated by different colors, and “others” represent the remaining members. (a) 14 samples were directly compared; (b) Comparison after grouping 14 samples into FN (foreign) and CN (China). [file 13568_2023_1580_MOESM1_ESM.pdf]

Analysis of differences in tobacco leaf microbial communities after redrying at home and abroad

Yifan Zhang [1], Qiang Xu [1], Mengmeng Yang[2], Yue Yang [1], Jincun Fu [1], Chenlin Miao [1], Guiyao Wang[2], Liwei Hu[2 *] and Zongyu Hu [1 *]

1 China Tobacco Jiangsu Industrial Co., Ltd., Nanjing 210000, Jiangsu, China

2 Zhengzhou Tobacco Research Institute of CNTC, Zhengzhou 450001, Henan, China

Yifan Zhang, Qiang Xu and Mengmeng Yang these authors contributed equally to this work.

* Corresponding author:

Liwei Hu, Zhengzhou Tobacco Research Institute of CNTC, Zhengzhou 450001, Henan, China.

Email: 229520489@qq.com

Zongyu Hu, China Tobacco Jiangsu Industrial Co., Ltd., Nanjing 210000, Jiangsu, China

Email: huzy707@sina.com

Supplementary Figure

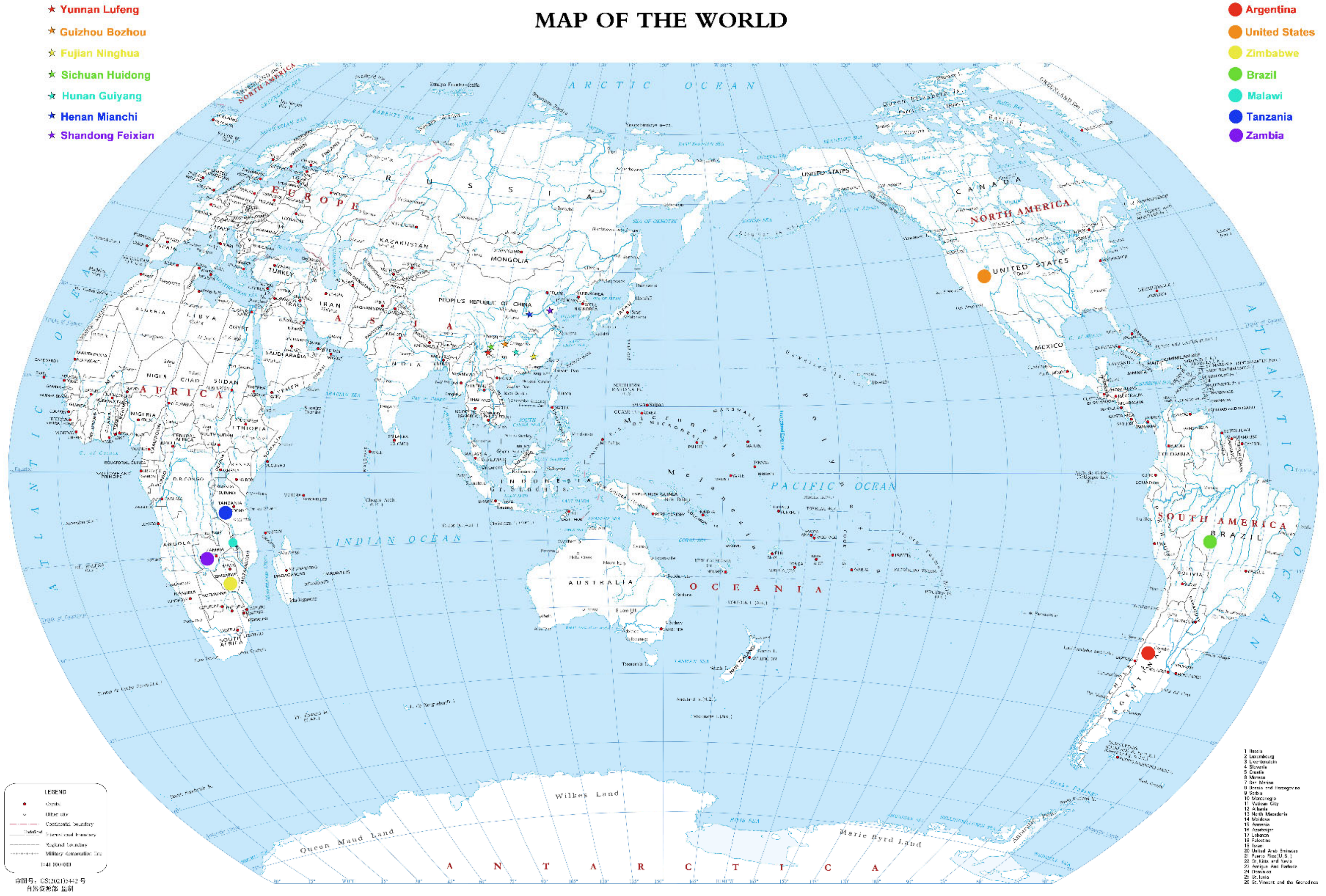

Supplementary Figure S1 The rough location of the tobacco leaf sample marked on the world map. The pentagram indicates the approximate location of domestic tobacco samples, and the dots indicates the approximate location of foreign tobacco samples.

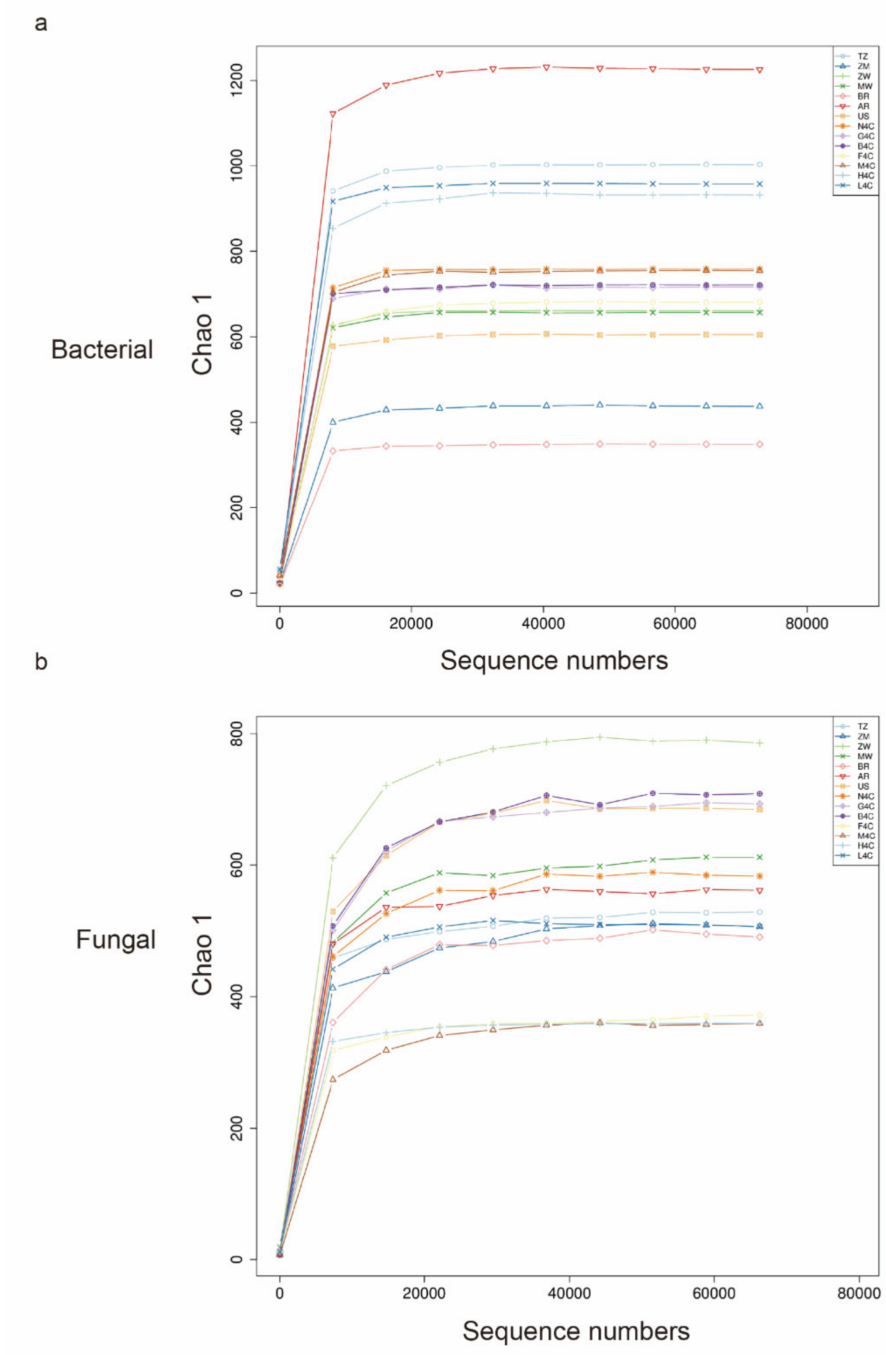

Supplementary Figure S2 Diversity and composition of bacterial and fungal communities in tobacco leaves after threshing and redrying. **a)** Chao richness values of bacterial communities **b)** Chao richness values of fungal communities

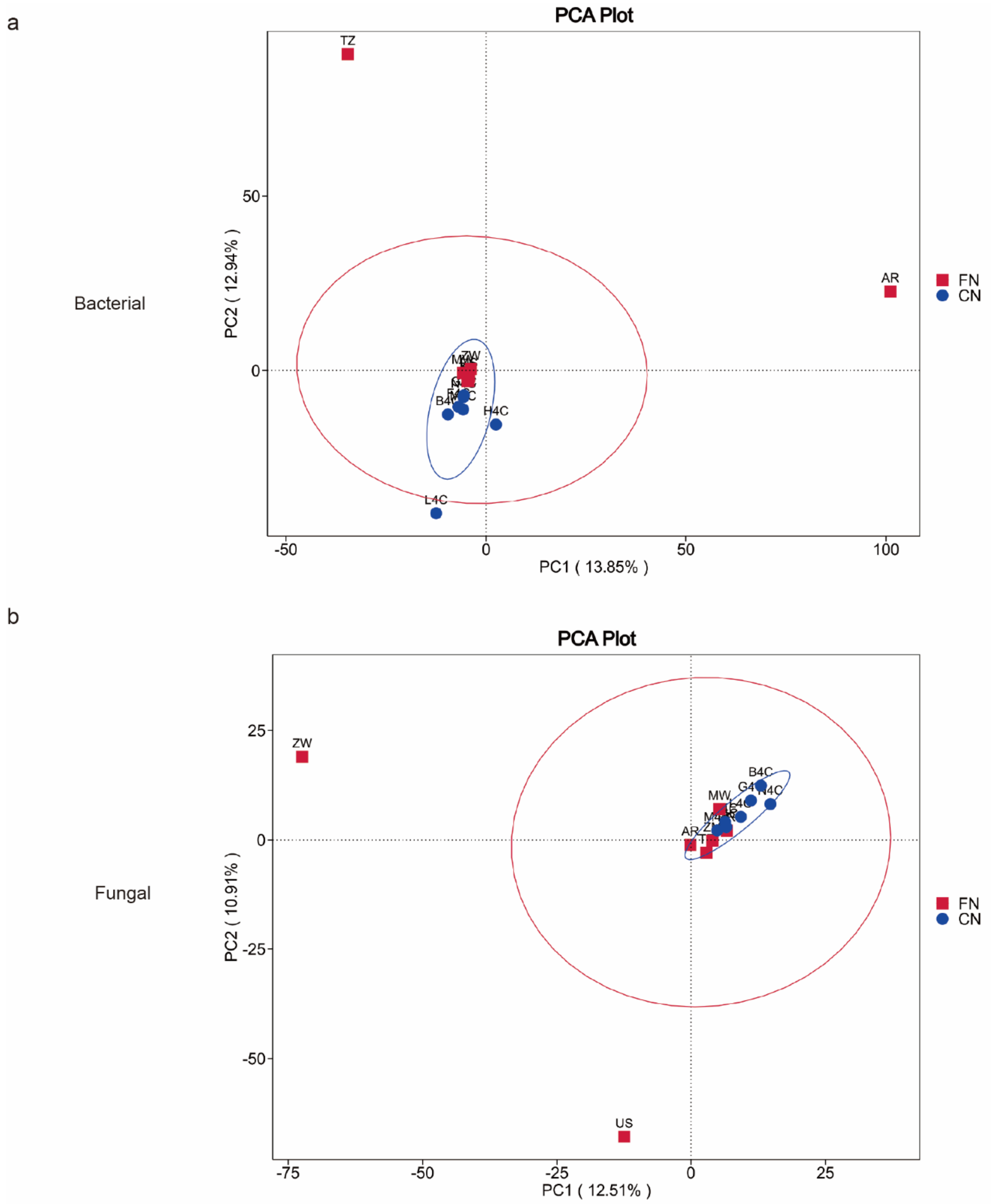

Supplementary Figure S3 PCA analysis of bacterial (a) and fungal (b) communities in 14 tobacco leaf samples at ASV level. (FN, foreign; CN, China; CN samples were indicated in blue in the figure and FN samples were indicated in red in the figure).

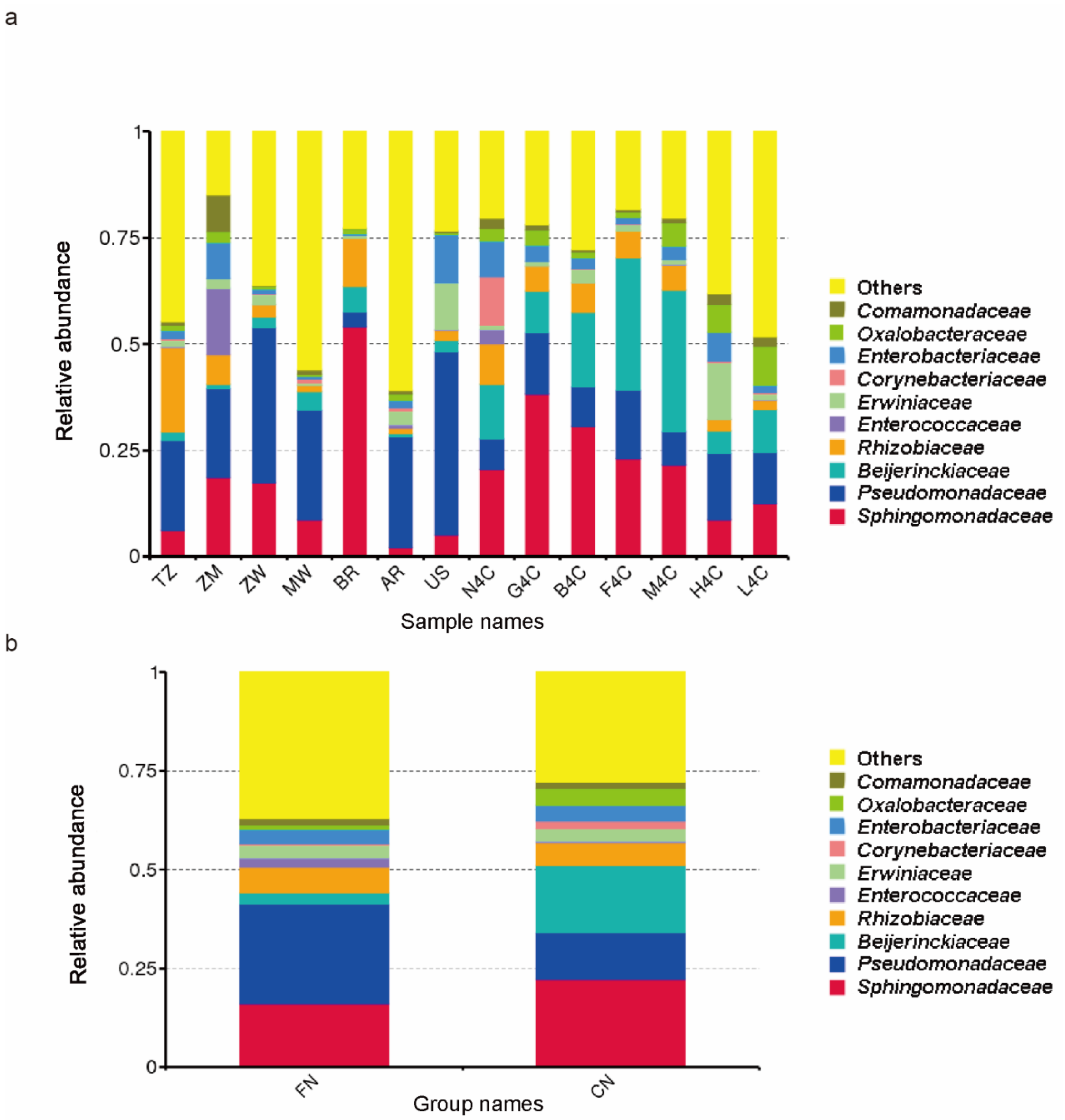

Supplementary Figure S4 Bacterial communities of tobacco leaf at family level. The top10 bacterial families are indicated by different colors, and “others” represent the remaining members. **(a)** 14 samples were directly compared; **(b)** Comparison after grouping 14 samples into FN (foreign) and CN (China).

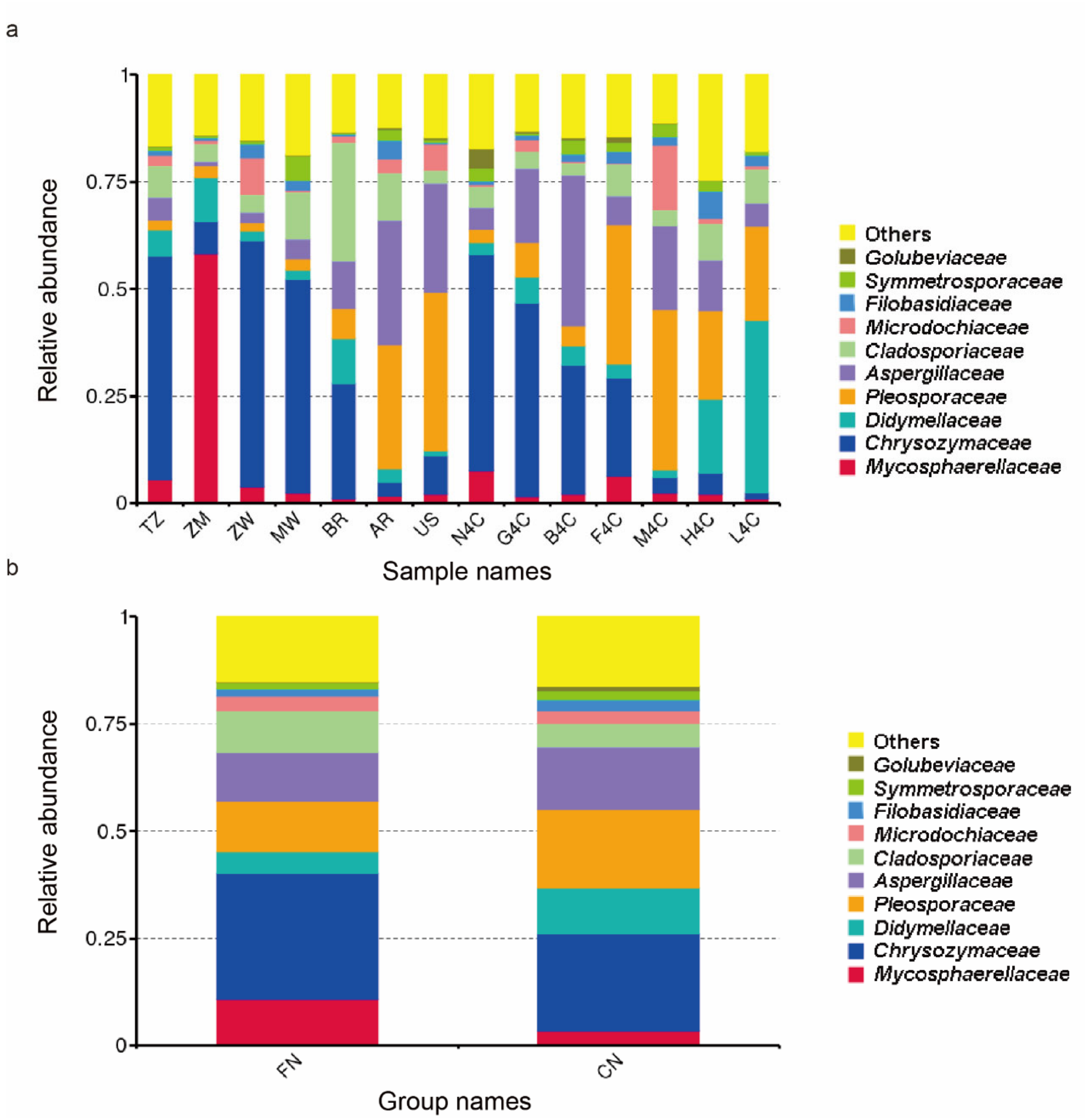

Supplementary Figure S5 Fungi communities of tobacco leaf at family level. The top10 fungi families are indicated by different colors, and "others" represent the remaining members. **(a)** 14 samples were directly compared; **(b)** Comparison after grouping 14 samples into FN (foreign) and CN (China).

Supplementary Tables
